# Imaging of Multifocal Adenoid Cystic Carcinoma of the Breast Initially Diagnosed as Adenomyoepithelioma on Core Needle Biopsy: A Case Report

**DOI:** 10.7759/cureus.116150

**Published:** 2026-09-12

**Authors:** Evan T Chandlee, Pratik Gongloor, Vedant Agrawal, Quan D Nguyen

**Affiliations:** 1 Department of Radiology, The University of Texas Medical Branch, Galveston, USA; 2 Department of Radiology, Baylor College of Medicine, Houston, USA

**Keywords:** adenoid cystic carcinoma, adenomyoepithelioma, atypical ductal hyperplasia, breast, breast mri, core needle biopsy, radiologic-pathologic correlation, stereotactic biopsy, triple-negative breast cancer

## Abstract

Adenoid cystic carcinoma (ACC) of the breast is a rare, typically triple-negative malignancy with an indolent course and a biphasic epithelial and myoepithelial histology that can be mistaken for benign or high-risk lesions on core needle biopsy. We describe a 52-year-old woman with two masses at the 4 o'clock position of the right breast, 5 cm and 7 cm from the nipple, in whom ultrasound-guided core needle biopsy of the anterior lesion yielded atypical ductal hyperplasia and stereotactic core needle biopsy of the posterior lesion yielded adenomyoepithelioma. Surgical excision of both sites revealed invasive ACC with foci measuring 24 mm and 4 mm, and after mastectomy without adjuvant therapy, the patient remained free of recurrence for 10 years. This case illustrates that ACC of the breast can be misclassified as atypical ductal hyperplasia or adenomyoepithelioma on core needle biopsy and underscores the importance of radiologic-pathologic correlation and surgical excision of high-risk or discordant lesions.

## Introduction

Adenoid cystic carcinoma (ACC) of the breast is a rare malignancy that accounts for approximately 0.1% of all breast carcinomas [1,2]. It most commonly affects women in the fifth to seventh decades of life [1,2]. Clinically, ACC of the breast often presents as a palpable mass, frequently in the subareolar region, and may be associated with tenderness or pain [2,3]. Imaging findings are non-specific. On mammography, ACC typically appears as a round, oval, irregular, or lobulated mass, often with obscured or indistinct margins and usually without suspicious calcifications, and on ultrasound it most often appears as an irregular hypoechoic mass [2]. Histologically, ACC closely resembles its salivary gland counterpart and consists of a biphasic proliferation of luminal epithelial and basal myoepithelial cells arranged in cribriform, tubular, or solid patterns [3].

Despite frequently demonstrating a triple-negative immunophenotype, with absent expression of estrogen receptor (ER), progesterone receptor (PR), and human epidermal growth factor receptor 2 (HER2), ACC of the breast typically follows an indolent clinical course and carries a favorable prognosis compared with other triple-negative breast carcinomas [1,3]. In a population-based cohort, 10-year relative survival exceeded 90%, most patients presented with localized disease, and axillary nodal involvement and distant metastasis at presentation were uncommon [1]. We present a case of invasive ACC of the breast involving two distinct lesions that were initially interpreted on core needle biopsy as atypical ductal hyperplasia (ADH) and adenomyoepithelioma (AME). This case highlights the diagnostic challenge posed by biphasic breast tumors, defined as neoplasms composed of two distinct cell populations, an inner luminal epithelial layer and an outer myoepithelial layer, recapitulating the normal two-cell architecture of the mammary duct. Because this dual population is preserved in benign lesions and lost in most conventional invasive carcinomas, its presence in a core sample may be misinterpreted as evidence against malignancy. This report underscores the importance of careful radiologic-pathologic correlation and surgical excision when percutaneous biopsy results do not fully account for suspicious imaging findings.

## Case presentation

A 52-year-old woman presented for diagnostic evaluation of the right breast. She reported intermittently feeling an irregularity in the breast over the preceding several years but denied nipple discharge, skin changes, or significant tenderness. Screening mammography performed several months earlier had demonstrated multiple masses in the lower inner quadrant of the right breast that had increased in size compared with prior examinations; spot compression views and ultrasound had been recommended, but the patient had not returned for follow-up. Her history was notable for prior abnormal mammograms attributed to cysts that had been aspirated years earlier. Her family history was notable for a sister diagnosed with breast cancer at age 42 years who died of metastatic disease. Physical examination did not reveal skin changes, nipple retraction, or a discrete palpable mass.

**Figure 1:**
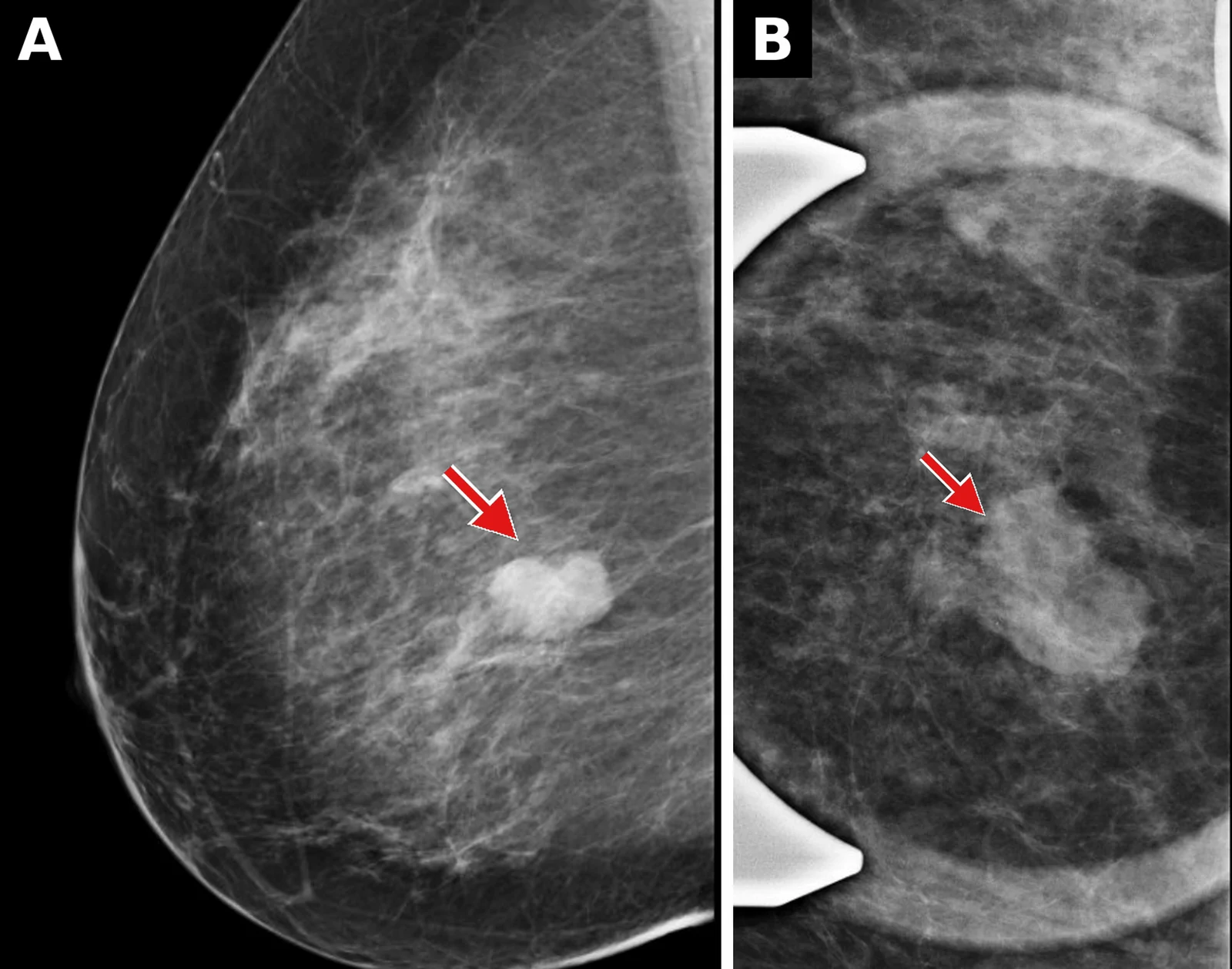
Diagnostic mammogram of the right breast. Mediolateral (A) and spot compression craniocaudal (B) views demonstrate an equal-density lobulated mass with obscured margins at the 4 o'clock position, approximately 5 cm from the nipple (red arrows), within heterogeneously dense breast tissue.

**Figure 2:**
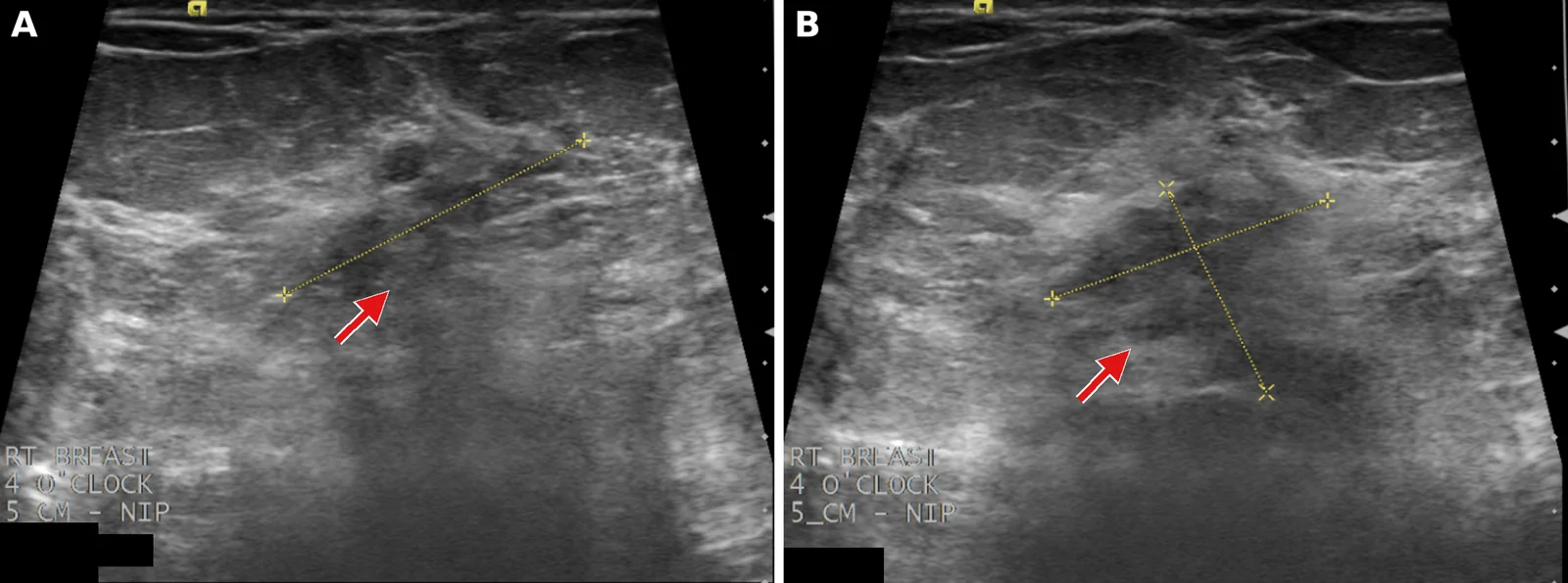
Targeted ultrasound of the right breast at the 4 o'clock position. Grayscale images in two orthogonal planes (A, B) at the 4 o'clock position, 5 cm from the nipple, demonstrate an irregular hypoechoic mass (red arrows) measuring 23x20x15 mm between the calipers, corresponding to the mammographic mass.

Diagnostic mammography demonstrated heterogeneously dense breast tissue and an equal-density lobulated mass with obscured margins at the 4 o'clock position of the right breast, approximately 5 cm from the nipple (Figures 1A, 1B). Targeted ultrasound of this region demonstrated a corresponding irregular hypoechoic mass measuring approximately 23x20x15 mm (Figures 2A, 2B). On the basis of these features, the lesion was assessed as Breast Imaging Reporting and Data System (BI-RADS) category 4B, and ultrasound-guided core needle biopsy was recommended [4]. A second mass at the 4 o'clock position, approximately 7 cm from the nipple, was identified mammographically, and tissue sampling of both sites was planned. Dedicated mammographic images of this second mass were unavailable for inclusion because of archiving limitations.

The anterior lesion, 5 cm from the nipple, corresponded to the hypoechoic mass identified on ultrasound and was sampled by ultrasound-guided core needle biopsy with a 14-gauge automated biopsy instrument (Tempe, AZ: Bard Biopsy Systems); six specimens were obtained, and a wing-shaped marker clip was placed at the biopsy site. Histopathologic examination demonstrated atypical ductal hyperplasia (ADH) with associated fibrocystic changes, including stromal fibrosis and usual ductal hyperplasia. The posterior lesion, 7 cm from the nipple, was sampled by stereotactic core needle biopsy with a 9-gauge vacuum-assisted biopsy device (Eviva; Marlborough, MA: Hologic, Inc.); four specimens were obtained, and a ribbon-shaped marker clip was placed. Histopathologic examination demonstrated a benign myoepithelial cell proliferation with a glandular component, consistent with adenomyoepithelioma (AME). The two biopsy sites were separated by approximately 7 cm.

**Figure 3:**
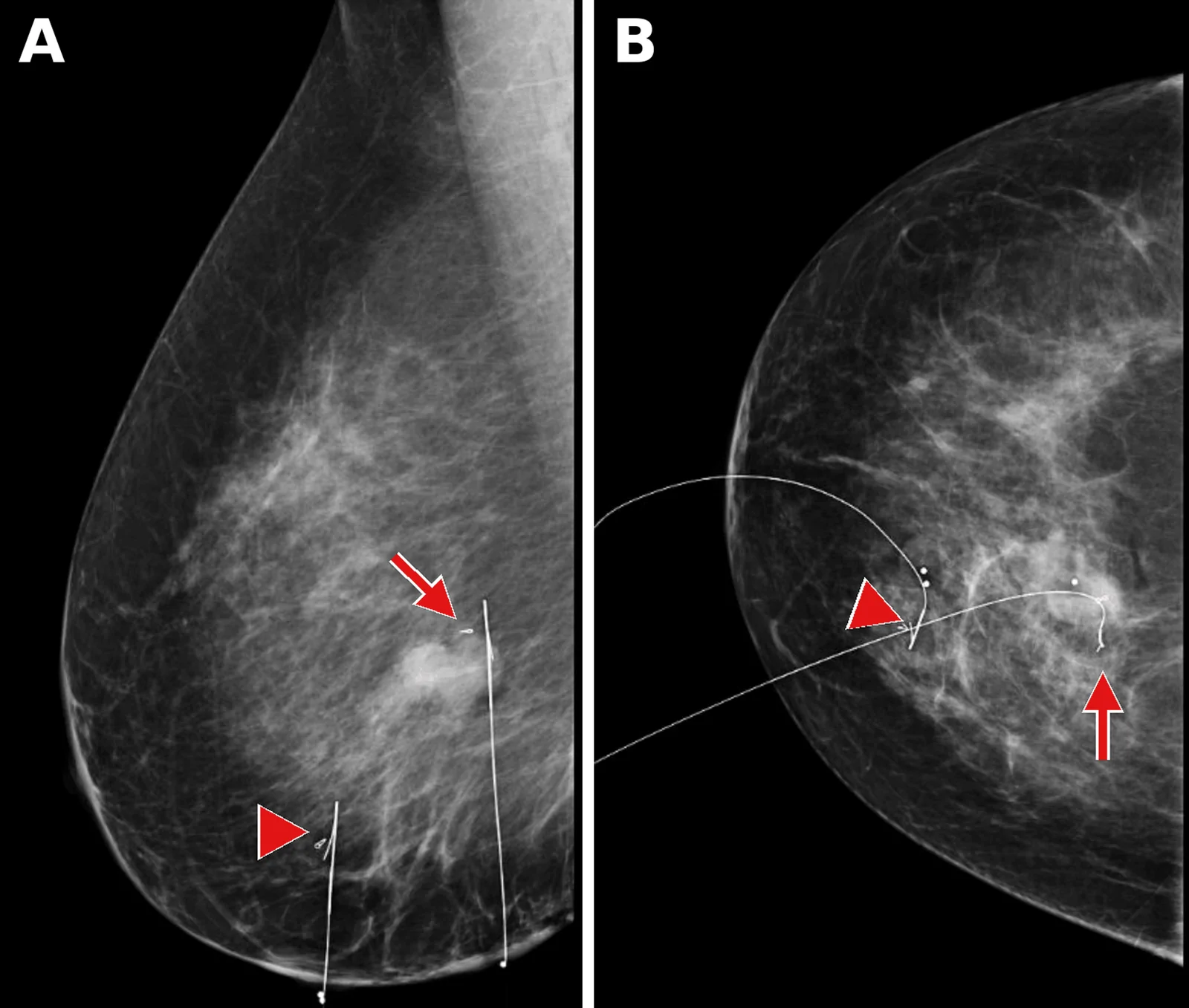
Mammographic wire localization of both biopsy sites. Mediolateral (A) and craniocaudal (B) views obtained after stereotactic wire localization demonstrate two hook wires with tips adjacent to the marker clips at the anterior biopsy site, 5 cm from the nipple (arrowheads), and the posterior biopsy site, 7 cm from the nipple (arrows).

Both biopsy results represented high-risk lesions with a recognized potential for upgrade to malignancy at excision, and after review by the institutional breast imaging panel, the patient was referred for surgical excision of both sites. Following stereotactic wire localization of both marker clips, the patient underwent wide local excision, and specimen radiographs confirmed that both clips were contained within the excised tissue (Figures 3A, 3B). Final surgical pathology revealed invasive ACC involving both biopsy sites, with tumor foci measuring 24 mm and 4 mm. The carcinoma demonstrated cribriform and solid growth patterns with central necrosis and was composed of dual epithelial and myoepithelial cell populations, with tumor focally present at the inked margins. Immunohistochemistry demonstrated a triple-negative phenotype, with absent expression of estrogen receptor (ER), progesterone receptor (PR), and human epidermal growth factor receptor 2 (HER2), positive staining for the basal markers p63 and cytokeratin 5/6 (CK5/6), and a Ki-67 proliferation index of 30%, consistent with ACC. Hematoxylin and eosin-stained and immunohistochemically stained histopathologic images were unavailable for inclusion because of archiving limitations.

**Figure 4:**
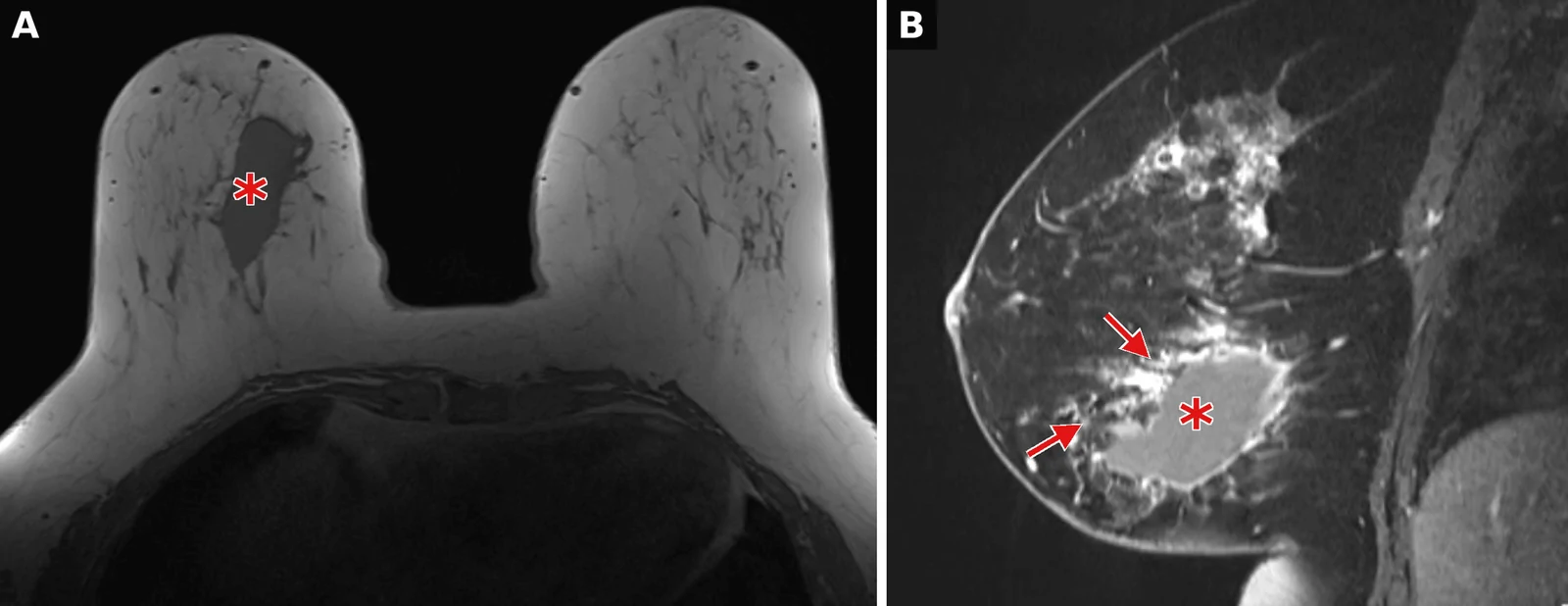
Postoperative breast MRI after wide local excision. (A) Axial T1-weighted image without fat suppression demonstrates a postoperative seroma in the right breast (asterisk). (B) Sagittal postcontrast T1-weighted fat-suppressed image of the right breast demonstrates the seroma (asterisk) with peripheral nodular enhancement along its anterior margin at the 5 o'clock position (red arrows), measuring 29x31x16 mm, consistent with residual tumor.

Sentinel lymph node biopsy was negative for metastatic disease, and the tumor was staged as pT2N0M0, stage IIA. Following multidisciplinary tumor board discussion, bilateral breast MRI was obtained to evaluate the extent of residual disease. MRI demonstrated a postoperative seroma measuring approximately 55x54x20 mm at the 6 o'clock position of the right breast. Along the medial and anterior aspect of the seroma, at the 5 o'clock position, there was peripheral nodular enhancement measuring 29x31x16 mm with rapid initial enhancement and delayed washout kinetics, consistent with residual malignancy and assessed as BI-RADS category 6 (Figures 4A, 4B). There was no evidence of additional multifocal or multicentric disease, and the left breast was negative. Re-excision was recommended, and the patient subsequently underwent right mastectomy without adjuvant chemotherapy or radiation therapy. Surveillance mammography during follow-up was negative (BI-RADS category 1), and she remained free of recurrence 10 years after surgery.

## Discussion

ACC of the breast is rare and, despite its triple-negative immunophenotype, carries a 10-year relative survival above 90% in population-based data, with most patients presenting with localized disease [1]. Its imaging features are non-specific as follows: mammography most often shows a round, oval, irregular, or lobulated mass with obscured or indistinct margins and no suspicious calcifications, and ultrasound shows an irregular hypoechoic mass, so ACC cannot be distinguished from other breast malignancies, or from some benign lesions, on imaging alone [2]. The findings in the present case fit this pattern and appropriately led to a Breast Imaging Reporting and Data System (BI-RADS) 4B assessment and biopsy of both lesions. Sampling each lesion under the imaging modality that best demonstrates it is standard practice and ensures accurate targeting [5].

Core needle biopsy yielded ADH at one site and AME at the other, both high-risk lesions. A meta-analysis of 6,458 percutaneously diagnosed pure ADH lesions reported a pooled upgrade rate of 29%, 23% with stereotactic and 42% with ultrasound guidance, and concluded that surgical excision should remain the standard of management [6]. AME is a rare biphasic tumor of epithelial and myoepithelial cells in which malignant transformation of either component is well documented and may be missed with limited sampling [7]; on imaging, it most often appears as an irregular mass with suspicious features, so excision is also the recommended management [8]. Excision of both sites in this case was therefore warranted on the biopsy results alone, and it revealed invasive ACC at both.

ACC and AME share dual epithelial and myoepithelial differentiation and overlapping histologic features [3,7]. Yang et al. described malignant AME and ACC coexisting within a single mass with a gradual histologic transition between them, supporting a shared epithelial-myoepithelial lineage [9]. When a core sample captures the myoepithelial-rich component of ACC predominantly, it may resemble a benign or borderline myoepithelial proliferation such as AME, leading to underestimation of malignancy [7,9]. The present case is unusual in that two adjacent lesions with different biopsy diagnoses proved to represent the same malignancy.

Distinguishing benign from malignant biphasic lesions rests on architectural and cytologic features rather than on the presence of a myoepithelial population alone. In most conventional invasive carcinomas, the myoepithelial layer is lost, so its retention in a biphasic lesion may be read as reassuring; in ACC, however, the myoepithelial-basal cells are an intrinsic component of the malignancy rather than a preserved normal layer [3]. Benign biphasic lesions such as AME retain an organized two-cell arrangement with circumscribed borders and low proliferative activity, whereas features favoring malignancy include infiltrative margins, cytologic atypia, necrosis, increased mitotic activity, and an elevated Ki-67 index [7]. On core needle biopsy, findings that should raise the possibility of ACC include cribriform or tubular architecture with pseudolumina, dual luminal epithelial and basaloid myoepithelial populations, and immunoreactivity for basal markers such as p63 and CK5/6 in the absence of ER, PR, and HER2 expression [3,9]. Because these features may be focal and the myoepithelial-rich component may dominate a small sample, a core diagnosis of a benign or borderline myoepithelial proliferation cannot reliably exclude ACC, and excision remains necessary when the imaging findings are suspicious [7,9].

Formal assessment of imaging-pathology concordance after every percutaneous biopsy is essential, and when the histology does not explain the imaging findings, repeat sampling or excision should follow [10]. Recognizing that ACC can mimic benign or high-risk lesions radiologically and histologically, along with multidisciplinary review of imaging and pathology, is the most reliable safeguard against delayed diagnosis [5,10].

Reported cases of ACC of the breast are summarized in Table 1. Across these reports, patients presented predominantly in the sixth and seventh decades, most often with a palpable mass, and imaging findings were consistently non-specific, with mammography showing a dense or irregular mass and ultrasound showing a hypoechoic mass. Management was surgical in every case, with breast conservation used most frequently and adjuvant radiotherapy applied variably. The present case differs in the following three respects: the abnormality was detected at screening without a discrete palpable mass, the disease was multifocal, and the diagnosis was established only after excision of two lesions whose core biopsies had both been non-malignant.

**Table 1:** Reported cases of adenoid cystic carcinoma of the breast. BI-RADS: Breast Imaging Reporting and Data System; NR: not reported; pT2N0: pathologic tumor stage T2, node negative; UOQ: upper outer quadrant

Studies	Age (years)	Presentation	Imaging findings	Management	Outcome
Canyilmaz et al. (2014) [11]	58	Pain in the right UOQ	Mammography: dense mass with spiculated extensions, BI-RADS 4	Breast conservation surgery with sentinel node dissection; adjuvant radiotherapy	No recurrence at 20 months
Yan et al. (2018) [12]	71	Painless right breast lump	Mammography: asymmetric density in the UOQ; ultrasound: 2 cm suspicious nodule	Surgery; adjuvant therapy declined by patient	Well at 6 months
Agafonoff et al. (2019) [13]	69	Tender, palpable left breast mass	Ultrasound: 2.2 cm heterogeneous hypoechoic mass; MRI: 5x3x2 cm mass	Breast-conserving therapy with sentinel lymph node biopsy; adjuvant radiotherapy	NR
Zhang et al. (2021) [14]	Median: 60.5 (range: 39-73), 14 patients	Palpable mass in 85.7%	Non-specific on mammography and ultrasound in all patients	Surgery in all patients	Single-institution series
Reyes et al. (2022) [15]	53	Palpable right breast mass	Mammography: 2 cm hyperdense mass with indistinct margins; ultrasound: bilobed 2.0x1.6 cm mass	Excision with sentinel lymph node biopsy (pT2N0)	Referred for adjuvant therapy; follow-up incomplete
Correa-Sandoval et al. (2025) [16]	56	Painless right breast lesion	Mammography and ultrasound: four multifocal nodules, BI-RADS 5	Bracket-guided partial mastectomy; adjuvant radiotherapy	No recurrence on short-term follow-up
Present case	52	Screening-detected; no discrete palpable mass	Mammography: lobulated mass with obscured margins; ultrasound: irregular hypoechoic mass, 23x20x15 mm, BI-RADS 4B; second mammographic mass	Excision of both sites, then mastectomy; no adjuvant therapy	No recurrence at 10 years

## Conclusions

Adenoid cystic carcinoma of the breast is a rare, typically triple-negative malignancy with an indolent course, non-specific imaging features, and a biphasic epithelial and myoepithelial histology that overlaps with benign and high-risk lesions. In this case, two adjacent suspicious lesions in the same quadrant were initially interpreted on core needle biopsy as atypical ductal hyperplasia and adenomyoepithelioma, and surgical excision revealed invasive adenoid cystic carcinoma at both sites. The finding of a benign or high-risk myoepithelial proliferation in a core sample from a suspicious mass should prompt consideration of adenoid cystic carcinoma, particularly when more than one lesion is present.

This case reinforces several principles of breast imaging practice. The biopsy technique should be matched to the modality that best demonstrates each lesion; every biopsy result should be formally assessed for concordance with the imaging findings, and high-risk or discordant results warrant surgical excision rather than imaging follow-up. Multidisciplinary review of imaging and pathology is the most reliable safeguard against underdiagnosis of rare biphasic breast tumors.
